# Reasons for Swearing as a Form of Self-Talk in Sport and Exercise: Development and Validation of a New Questionnaire

**DOI:** 10.3390/bs15050593

**Published:** 2025-04-28

**Authors:** Noam Manor, Gershon Tenenbaum

**Affiliations:** 1 Department of Physiotherapy, Faculty of Health Sciences, Ariel University, Shomron 407000, Israel; gtenenbaum@admin.fsu.edu; 2 B. Ivcher School of Psychology, Reichman University, Herzliya 4610101, Israel

**Keywords:** self-talk, swearing, sport, exercise, aggression, profanity

## Abstract

Swearing is a powerful and emotionally charged form of language that has recently gained increased scholarly attention. While commonly used for emotional release, its role in sport and exercise ‘self-talk’ remains understudied. Prior evidence suggests that swearing may aid in pain management, emotion regulation, and performance enhancement, yet research in athletic contexts has largely focused on conventional self-talk. To address this gap, the present study developed and validated the Reasons for Swearing in Sport and Exercise Questionnaire (RSSEQ), a novel tool assessing reasons motivating swearing among competitive athletes, coaches, and recreational exercisers. A total of 513 participants completed the RSSEQ. Exploratory (n = 333) and confirmatory (n = 180) factor analyses supported a three-factor structure: Stress and Emotional Catharsis, Mental Strength Enhancement, and Coping with Physical Discomfort and Pain. Males reported greater use of swearing for mental strength and pain-related coping, while no gender differences were observed for emotional catharsis. Athletes reported more swearing for emotional catharsis than did coaches, but no differences emerged in motivational or pain-related use. No significant differences were found between competitive and recreational athletes or between team and individual sport participants. These findings establish a foundation for future research on the psychological functions of swearing in sport and exercise, encouraging further exploration of its effectiveness and potential integration into mental training strategies, alongside more traditional self-talk approaches.

## 1. Introduction

Language shapes human cognition, emotion, and behavior (Lindquist et al., 2015; Lindquist, 2017; Lupyan & Clark, 2015). Among the various forms of language, swearing stands out as a unique linguistic tool, often dismissed as inappropriate or unprofessional, yet increasingly recognized for its psychological and physiological effects (Stapleton et al., 2022). Research findings revealed that swearing modulates acute pain (Hay et al., 2024) and increases physical performance (Washmuth et al., 2024), both of which are crucial factors in the sport domain. Despite these potential benefits, swearing remains underexplored in sport and exercise psychology, particularly in relation to self-talk (ST), which is defined as “verbalizations or self-statements addressed to the self” (Hardy, 2006, p. 84). Recognizing the motivations which underly the act of swearing in sport and exercise contexts is therefore crucial for expanding our perspective on ST as a psychological strategy. Consequently, our objective is to develop and validate a questionnaire that is psychometrically sufficiently strong to effectively capture this phenomenon.

### 1.1. Swearing: Definition and Functions

Swearing refers to “the use of words which have the potential to be offensive, inappropriate, objectionable, or unacceptable in any given social context” (Beers Fägersten, 2012, p. 3). It is a universal linguistic phenomenon found across cultures, languages, and historical periods (Montagu, 2001; Van Lancker & Cummings, 1999; Vingerhoets et al., 2013). Although swearing is often considered taboo, the term “swearing paradox” describes the phenomenon in which individuals frequently use offensive language in everyday interactions despite its negative connotations (Beers Fägersten, 2012). Similarly, Rosenberg et al. (2017) and Jay (2018) argued that the most offensive words are often among the most used, further reinforcing this paradox.

In the 15th century, swearing could lead to various penalties, including death (Pinker, 2007; Stone & Hazelton, 2008). However, swearing is now recognized as a distinct form of linguistic activity, fundamentally different from other types of language (Stapleton, 2010), and has been described as “powerful” (Stapleton et al., 2022, p. 2). Montagu (2001) differentiated between *social swearing* and *annoyance swearing.* Social swearing fulfills social functions like fostering bonds, injecting humor, and expressing group identity, typically in casual or close-knit settings. Conversely, annoyance swearing is intrapersonal; it acts as an immediate emotional release in response to frustration, pain, or stress, usually occurring in private rather than social situations. This study specifically examines annoyance swearing as a possible method for emotional regulation in sports and exercise contexts. Moreover, swearing was found to enhance pain tolerance, raise pain thresholds, and diminish perceived pain (Hay et al., 2024).

A review by Washmuth et al. (2024) summarized the existing evidence on the effects of swearing on physical performance, further supporting claims of its performance-enhancing benefits across various physical tasks (e.g., handgrip strength, mean and peak power output during anaerobic tests, plank, push-ups, and wall sit). Their findings suggest that swearing may be an accessible and immediate strategy for the enhancement of physical performance and muscular strength and endurance, which may be particularly relevant for individuals engaged in sport and exercise. However, both reviews emphasize that while swearing produces positive effects across different contexts and tasks, the underlying mechanisms behind this effect remain unclear, warranting further investigation.

Additional research findings revealed that repeating a swear word, as opposed to a neutral word, enhances performance on a grip strength task (Beck et al., 2025). This research also confirmed the effects of swearing on positive emotions, humor, and distraction. Moreover, swearing during an endurance task helped prevent mood deterioration following intense exercise (Ballmann et al., 2024). While it did not enhance endurance performance compared to a neutral word condition, it provided psychological benefits by counteracting the decline in total mood disturbance observed in the neutral condition. These findings suggest that swearing is particularly beneficial for recreational exercisers, as a more positive psychological experience during workouts may enhance adherence to exercise regimens (Rhodes & Kates, 2015). Thus, integrating swearing as a strategy to improve workout perception may support sustained engagement in physical activity.

Recently, Adebiyi et al. (2025) studied the effects of swearing and yelling on exercise performance and pain tolerance in 20 American (U.S.) and 20 Thai participants, who underwent five conditions: control, self-yelling, observer-yelling, self-swearing, and observer-swearing while completing a handgrip strength test, plank exercise, and cold pressor test. Among U.S. participants, swearing (both self- and observer-generated) significantly improved plank time compared to the control, and observer-swearing increased handgrip strength. Yelling failed to affect performance. In Thai participants, self-yelling increased handgrip strength, and observer-yelling prolonged cold pressor test time, but neither yelling nor swearing affected plank time. These findings suggest cultural and contextual variations in the efficacy of swearing and yelling as performance-enhancing strategies. Overall, the findings revealed that swearing represents a promising strategy for the enhancement of physical performance, regulating pain sensations, and influencing the psychological states which affect motor performance. These benefits make it a highly valuable tool in sports and exercise settings, in which the maximizing of strength, endurance, and pain tolerance is essential for achieving peak performance.

### 1.2. Self-Talk: Prevalence, Forms, and Effects

According to Winsler et al. (2006), 96% of adults engage in ST, while Nedergaard et al. (2021) found that over 85% of athletes use ST during sports, with the majority believing it enhances their performance. Given its widespread use, it is unsurprising that ST has been a central topic in sport psychology since the 1970s (Williams & Straub, 2006) and is now recognized as a well-established, extensively researched strategy for improving physical performance across various tasks and populations (Dali & Parnabas, 2018; Galanis et al., 2022; Hatzigeorgiadis et al., 2011; Tod et al., 2011, 2015; Young et al., 2023).

Hardy et al. (2008) proposed a multidimensional conceptual framework for ST, emphasizing its influence on cognitive (attention, focus), motivational (confidence, persistence), affective (emotional regulation), and behavioral (technique, execution) mechanisms. Alongside Hardy’s model, Van Raalte et al. (2016) introduced a sport-specific ST model that consists of a dual-process theory of cognition (Kahneman, 2011) which distinguishes between System 1 (automatic, emotionally driven ST) and System 2 (deliberate, controlled ST). Their model describes how ST affects cognitive and affective mechanisms (e.g., confidence, anxiety regulation) as well as behavioral outcomes (e.g., motor execution, persistence, and task disengagement). Despite these contributions, existing models of ST fail to account for unique forms of language, such as swearing, within cognitive, motivational, affective, and behavioral mechanisms. To our knowledge, no research has incorporated swearing into these conceptual frameworks, leaving its psychological functions in sports and exercise largely unexplored. This study addresses this gap by systematically examining how swearing functions within ST models, enhancing their ecological validity and enabling a more precise scientific investigation of its role in emotion regulation, performance enhancement, and psychological adaptation.

ST is often divided into organic and strategic forms (Latinjak et al., 2014). Organic ST occurs naturally and can be further divided into spontaneous (e.g., unplanned, reflexive utterances) and goal-directed (e.g., purposeful, though not formally structured) ST. Swearing typically correlates with goal-directed ST, acting as a quick emotional reaction to feelings of frustration, pain, anger, or sadness (Dewaele, 2004; Jay & Janschewitz, 2008). These reactions enable individuals to manage their feelings more effectively (Popuşoi et al., 2018). Therefore, swearing operates as a psychological mechanism that supports emotional regulation. In contrast, strategic ST is deliberately planned and structured, often used as part of a mental skills training program employed to achieve specific performance outcomes. Notably, all studies conducted to date that have investigated the effects of swearing on physical performance and pain sensation have regarded it as a strategic form, encouraging participants to swear intentionally when experiencing pain, such as during a cold pressor task, or before engaging in physical activity. Additionally, ST is traditionally classified into positive and negative ST, though it recently has been assigned alternative conceptual frameworks, such as *facilitating* (i.e., enhancing performance) or *debilitating* (i.e., hindering performance), to better capture its functional impact (Latinjak et al., 2023). Swearing, despite often being classified as a negative language, does not necessarily have a debilitating effect. Instead, research findings showed that it can function as a facilitating form of ST, contributing to performance enhancement, pain tolerance, and emotional regulation (Hay et al., 2024; Stapleton et al., 2022; Washmuth et al., 2024). Another distinction commonly has been made between motivational and instructional ST. Motivational ST is typically used to boost effort, confidence, and persistence, while instructional ST focuses on technique, strategy, and task execution (Theodorakis et al., 2000). Swearing seems to align more with motivational ST, as it is often used to enhance intensity during physically and mentally demanding moments (Stephens et al., 2018). Unlike traditional motivational ST (e.g., “You got this!”), swearing adds an emotional charge that may provide an additional psychological boost, especially when experiencing high-intensity exercise. According to Hardy (2006), ST can be categorized by overtness; specifically, it can take various forms, including being spoken aloud, mouthed without vocalization, or entirely internalized (Hardy, 2006; Oppenheim & Dell, 2010). Although research on the topic is limited, findings suggest that overt and covert ST can enhance performance, with varying degrees of effectiveness based on individual personality traits (Hong et al., 2020). Specifically, covert ST appears to be more beneficial for introverts, while overt ST is more advantageous for extroverts, indicating that the mode of ST may need to be tailored to an athlete’s personality orientation for optimal performance outcomes. Swearing, which is typically overt, aligns with findings that individuals are more likely to verbalize negative ST (Van Raalte et al., 1994). Given that extroverts may benefit more from overt ST, swearing might serve as a particularly effective self-regulatory tool for these individuals.

In sum, ST, particularly its facilitative aspect (as opposed to deliberative), is a powerful psychological tool that enhances physical performance, emotional regulation, and focus, making it especially valuable in sports and exercise settings. It serves multiple functions, including boosting motivation and concentration (Hatzigeorgiadis & Galanis, 2017; Wallace et al., 2017), increasing confidence (Walter et al., 2019), and managing stress (Kross et al., 2014), while also aiding in coping with adversity, and allowing athletes to excel under pressure (Hufton et al., 2024). Within this conceptual framework, swearing is considered a unique, yet overlooked, form of ST, offering additional psychological benefits beyond conventional language (Stapleton et al., 2022). Whether spontaneous or strategic, swearing may play a critical role in optimizing mental and physical performance and should be further investigated as a potential performance-enhancing tool in the sport and exercise psychology domain.

### 1.3. The Present Study

Van Raalte et al. (2016) noted that much ST research has focused on motivational and instructional ST. Thus, different types of language must be used and studied when investigating questions such as how swearing can be incorporated to induce motivation and concentration during or before physical exertion. Studies have predominantly examined traditional motivational ST (e.g., “You can do it!”) in comparison to neutral words or no guidance, yet this approach may fail to capture the diverse and dynamic ways individuals use language in real-world settings, particularly under adversity. Since repeating a swear word was found to be highly beneficial for physical performance, compared to repeating a neutral word, it is plausible that swearing can outperform traditional motivational ST cues. Furthermore, while athletes may engage in conventional ST outside laboratory settings, they are likely to incorporate swearing as part of their self-directed speech in real-life scenarios. However, if swearing remains understudied, its potential psychological and performance benefits may be overlooked.

One of the primary functions of swearing is to vent emotions and provide emotional release (Vingerhoets et al., 2013), suggesting that it may play a unique role in helping individuals cope with stress and adversity. Given the high-pressure nature of competitive sports and the physical and mental demands of exercise, exploring whether alternative forms of language can offer additional benefits compared to traditional and more conservative ST is pivotal. Within this framework, we propose that swearing may function as a form of motivational ST, particularly in contexts requiring increased effort or pain tolerance, or when ‘psyching up’ before stressful or challenging moments. In other words, we hypothesized that one function that swearing serves in athletic settings is to boost motivation before and during events. Given this, it is now timely to investigate the use of swearing in more depth, following the finding of Van Raalte and Vincent (2023) that athletes may benefit from finding the “right things” to say to themselves to maximize performance. However, developing a validated and reliable tool to assess the motivations behind swearing in sports and exercise settings is essential before evaluating the effectiveness of the practice.

While research investigating the general population suggests that swearing is primarily used for emotional regulation and stress management (Stapleton et al., 2022), its application in athletic contexts may involve additional or distinct motivations. To address this gap, the present study examines the reasons athletes, coaches, and recreational exercisers use swear words in sport and exercise, considering differences in competitive level (i.e., competitive athletes and coaches vs. recreational exercisers), professional role (i.e., athletes vs. coaches), sport type (i.e., team vs. individual), and gender (i.e., males vs. females). By comparing these groups, this study aims to provide a comprehensive conceptual framework of the motivations underlying swearing as a form of ST, laying the groundwork for future research on its potential applications in performance enhancement and emotional regulation. To reach this goal, we first developed a questionnaire designed to examine the relationship between situational triggers and swearing as a form of ST in sport and exercise and subsequently validated it.

## 2. Materials and Methods

### 2.1. Initial Sampling and Procedure for Items’ Generation

The study took place between May 2023 and July 2024. To compile a comprehensive list of reasons for the use of swear words among athletes and coaches during both training and competition, a sample of 15 professional athletes and coaches was recruited. They were aged *M* = 33.07, *SD* = 11.98 years, representing both males (n = 11) and females (n = 4) from different sporting disciplines (basketball n = 4, soccer n = 5, modern pentathlon n = 1, Olympic weightlifting n = 1, swimming n = 1, powerlifting n = 2, bodybuilding n = 1, mixed martial arts n = 1). Recruitment efforts included social media advertising, direct outreach to athletes receiving mental services, and colleague referrals. Participants were engaged in structured phone interviews to acquire representative data on swearing behaviors in sports, each lasting approximately 10 min. Before beginning the interview, participants were informed about the study’s objectives and assured of the confidentiality of their responses. During the interview, participants were asked the following question: “During training and competitions, do you personally use any swear words? If yes, please indicate the reason you use swear words during training and competitions.” To ensure thorough data collection, we also asked, “Are there any other reasons you might swear as a part of your ST?” and provided examples of challenging scenarios in training and competition that might prompt a swearing response. The responses from the initial inquiry guided the development of the Reasons for Swearing in Sport and Exercise Questionnaire (RSSEQ). A final list of 26 reasons was developed after the data collection and analysis process (see Table 1). Two sport and exercise psychology specialists reviewed these items, one holding a PhD and the other a professorial position, and both agreed on the final list. The responses from the initial inquiry guided the development of two instruments: the Use of Swear Words in Sport and Exercise Questionnaire (USWSEQ) and the RSSEQ. Due to space constraints, the development process of the USWSEQ is reported separately.

### 2.2. Data Generation

Participants were recruited through social media advertisements; by email outreach to sport organizations, aiming to engage their athletes and coaches; and by asking peers who work with athletes to distribute the questionnaires. To recruit participants effectively, an incentive was provided: all participants were offered entry into a lottery, with three winners receiving a spa package. Data collection was facilitated using the Google Forms platform. The sample comprised active and retired competitive coaches, athletes, and recreational exercisers. Participants were recruited from various competitive backgrounds, representing different levels of competition. Competitive athletes and coaches were eligible for inclusion if they had competed within the past 12 months in a recognized league or tournament at the regional, national, or international level. This criterion, which included involvement in professional leagues, major national championships, or internationally sanctioned events, ensured they maintained their experience in structured, competitive environments.

Moreover, recreational exercisers were included if they had adhered to an exercise regimen for at least 6 months, training at least twice per week, without competing in formal leagues or tournaments. For retired athletes and coaches, inclusion consisted of declared previous competitive or coaching involvement at a recognized level (e.g., regional, national, or international levels). Although they were no longer actively competing or coaching, these participants indicated past engagement in structured, competitive sports, aligning them with the profiles of current athletes and coaches regarding professional experience. The diverse participant pool provided a comprehensive dataset which could be used for conducting exploratory factor analysis (EFA), ensuring that the findings would reflect a broad spectrum of athletic experiences and recreational exercisers.

Ultimately, 333 participants were recruited (*Mage* = 32.8, *SD* = 12.7), including 172 females (51.6%), 157 males (47.2%), and 4 participants (1.2%) who selected “prefer not to say”. Of these, 156 (46.8%) were competitive athletes or coaches, and 177 (53.2%) were recreational athletes; 147 (44.1%) held a high school diploma, and 98 (29.4%) had earned a bachelor’s degree, 61 (18.3%) a master’s degree, and 1 (0.3%) a PhD, while 26 (7.8%) reported “other”. A total of 140 (42.0%) reported more than 10 years of professional experience, 83 (24.9%) 6–10 years, 77 (23.1%) 3–5 years, 28 (8.4%) 1–2 years, and 5 (1.5%) reported less than one year. Of these, 24 (7.2%) trained more than 20 h per week, 36 (10.8%) 16–20 h, 69 (20.7%) 11–15 h, 147 (44.1%) 5–10 h, and 57 (17.1%) trained fewer than 5 h per week.

### 2.3. Instrumentation

Demographic questionnaire (DM). The DM was designed to gain information about demographic variables such as age, gender, nationality, educational background, primary sport of interest, weekly hours of training, and years of experience.

Reasons for Swearing in Sport and Exercise Questionnaire (RSSEQ). Participants were given a list of 26 reasons and asked to rate the extent to which they agreed with each reason with respect to their ST. Responses were made on a Likert-type scale ranging from 1 (not at all) to 5 (to a very great extent).

The Competitive Aggressiveness and Anger Questionnaire (CAAS; Maxwell & Moores, 2007). The CAAS is a 12-item self-report questionnaire designed to measure athletes’ perceived aggressiveness and anger during athletic competitions. The CAAS is partitioned into two 6-item subscales: one for anger and one for aggressiveness. It employs a 5-point Likert-type scale ranging from 1 (almost never) to 5 (almost always) for responses. A sample item from the anger subscale reads as follows: “I find it difficult to control my temper during a match”. A sample item from the aggressiveness subscale reads as follows: “It is acceptable to use illegal physical force to gain an advantage”. Confirmatory factor analysis conducted by the authors indicated robust internal consistencies for the subscales and the overall scale score, including Cronbach’s alpha coefficients for anger (α = 0.78), aggressiveness (α = 0.84), and the total scale (α = 0.87).

The Brief Aggression Questionnaire (BAQ; Webster et al., 2014). The BAQ is a 12-item self-report measure designed to assess trait aggression, derived from the Buss–Perry Trait Aggression Scale (Buss & Perry, 1992). Participants are asked to rate the extent to which various statements reflecting behaviors and emotions are characteristic of them on a 5-point Likert-type scale ranging from 1 (strongly disagree) to 5 (strongly agree). The BAQ measures aggression across four sub-dimensions: physical aggression (e.g., “given enough provocation, I may hit another person”), verbal aggression (e.g., “I tell my friends openly when I disagree with them”), hostility (e.g., “other people always seem to get the breaks”), and anger-related emotion (e.g., “I have trouble controlling my temper”). Scores for these dimensions are averaged to yield a global score, with higher scores indicating higher levels of trait aggression. The BAQ is a valid and reliable measure, demonstrating strong and significant temporal stability, and ranging from 0.68 to 0.80 across the four subscales while reaching 0.81 for the overall BAQ (Webster et al., 2015). It also demonstrates robust convergent validity with established measures of aggression, including strong correlations, ranging from r = 0.75–0.85, with the Buss–Perry Aggression Questionnaire (Webster et al., 2014). Cronbach’s alpha reliability of the complete questionnaire in the current study was 0.71.

### 2.4. Procedure

Before data collection, ethics clearance was obtained from Ariel University’s IRB committee (Confirmation Number: AU-HEA-GT-20240307). Participants were informed about the purpose of the study, assured of their anonymity and confidentiality, and provided informed consent before proceeding. Data were collected electronically using Google Forms, with participants completing a demographic questionnaire, followed by the RSSEQ. Subsequently, the aggression-related measures were administered: the CAAS and the BAQ were given to athletes and coaches, while recreational exercisers completed only the BAQ. This research employed AI-enabled tools to generate and refine text. In particular, we used OpenAI’s ChatGPT (version 7 March, OpenAI, 2025, San Francisco, CA, USA) to improve clarity, grammar, and coherence during the writing process. However, the use of the AI tool was kept to a minimum, and it was not utilized for data analysis or hypothesis development.

### 2.5. Data Analysis

The sample size for this phase adhered to empirical recommendations for factor analysis (De Vellis, 2003; Lakens, 2022), ensuring sufficient statistical power and robust data analysis. An exploratory factor analysis (EFA) was conducted to determine the RSSEQ factor structure. The analysis utilized maximum likelihood estimation followed by Oblimin rotation. The suitability of the data was assessed by using the Kaiser–Meyer–Olkin (KMO) measure of sampling adequacy and Bartlett’s Test of Sphericity (BTS). Factors were retained based on the criterion of eigenvalues > 1.0. The EFA was performed on the 26 items of the initial RSSEQ to explore its underlying factor structure.

## 3. Results

The initial EFA revealed a four-factor structure accounting for 63.53% of the variance. The KMO measure of sampling adequacy was 0.94, and BTS was significant, *χ*^2^(325) = 6367.31, *p* < 0.001, indicating that the items maintained common variance sufficient for factor analysis. Item S25 was removed due to cross-loadings with factors 1 and 4, while item S26 was removed due to cross-loadings with factors 1 and 2. Factor 4 consisted of two items and was therefore eliminated. A second EFA was conducted on the remaining 24 items, revealing a three-factor structure accounting for 61.18% of the variance. The KMO measure of sampling adequacy was 0.94, and BTS was significant, *χ*^2^(276) = 5818.02, *p* < 0.001. The S21 cross-loaded on factors 1 and 2 and therefore was removed. A third EFA was conducted on the remaining 23 items. The KMO measure of sampling adequacy was 0.94, and BTS was significant, *χ*^2^(253) = 5509.38, *p* < 0.001. Item S19 had a factor loading of 1.005. Given its theoretical importance and the absence of significant statistical concerns (e.g., no extreme communalities or multicollinearity), it was retained for further analysis in the confirmatory factor analysis (CFA) to assess its stability within the overall model. No other items were cross-loaded. All retained items exceeded the recommended loading threshold of 0.50 (Fabrigar et al., 1999). The three factors were defined as follows: (1) Stress and Emotional Catharsis (Factor 1; eleven items)—reflects the use of swearing to release frustration, vent negative and intense emotions, and manage stress. This factor encompasses expressions aimed at alleviating anger, reducing tension, coping with disappointment, and channeling emotional intensity to maintain focus and composure during critical moments in training and competition; (2) Mental Strength Enhancement (Factor 2; ten items)—refers to swearing used as a psychological tool to strengthen confidence, sustain motivation, and enhance mental readiness. It involves verbal expressions that foster resilience, sharpen focus, regulate emotions, and optimize cognitive functioning to support peak performance, especially under stressful or challenging moments; and (3) Coping with Physical Pain and Discomfort (Factor 3; three items)—involves using swearing as a mental strategy to withstand pain, manage physical strain, and sustain performance in sports. This third factor includes verbal expressions that aid in pain tolerance, divert attention from discomfort, and reinforce mental toughness in the face of injuries or physical challenges. The results of the second EFA are presented in Table 2. The final structure provides a solid confirmatory factor analysis (CFA) foundation.

### 3.1. Sampling and Procedure

The CFA aimed to test the factor structure established by the EFA, verifying the reliability and validity of the identified three-factor structure of reasons for swearing. In sum, 180 participants were included in the CFA procedure (*Mage* = 32.28 years, *SD* = 11.01), of whom 82 (45.8%) were female, 96 (53.6%) were male, and 2 (0.6%) were unspecified. Of these, 87 (48.6%) were competitive athletes, coaches, or retired competitive individuals; 92 (51.4%) were recreational athletes; and one (0.6%) was unspecified. The highest-reported level of education of the participants was high school, with ninety-two (51.1%); followed by BA, with sixty-four (35.6%); others, eight (4.4%); and PhD, sixteen (8.9%). A total of 60 competitive athletes and coaches reported participating in individual sports, while 26 reported participating in team sports. We followed an identical protocol to that performed in the EFA stage, including similar incentives, to maintain consistency and ensure comparability of results.

### 3.2. Statistical Analysis

CFA was conducted to evaluate the measurement model. Reliability was assessed using Cronbach’s alpha, with values exceeding the recommended threshold (α > 0.70; Nunally, 1978). Composite Reliability (*CR*) (≥0.70) and Average Variance Explained (AVE) (≥0.50) were also calculated to further evaluate construct reliability and convergent validity. Model fit was evaluated using several statistical indices in line with Kline’s (2015) guidelines: Chi-square to degrees-of-freedom ratio (*CMIN/df* ≤ 3), Comparative Fit Index (*CFI* ≥ 0.90), Tucker–Lewis Index (*TLI* ≥ 0.90), Root Mean Square Error of Approximation (*RMSEA* ≤ 0.08), and Standardized Root Mean Square Residual (*SRMR* < 0.08).

**Figure 1:**
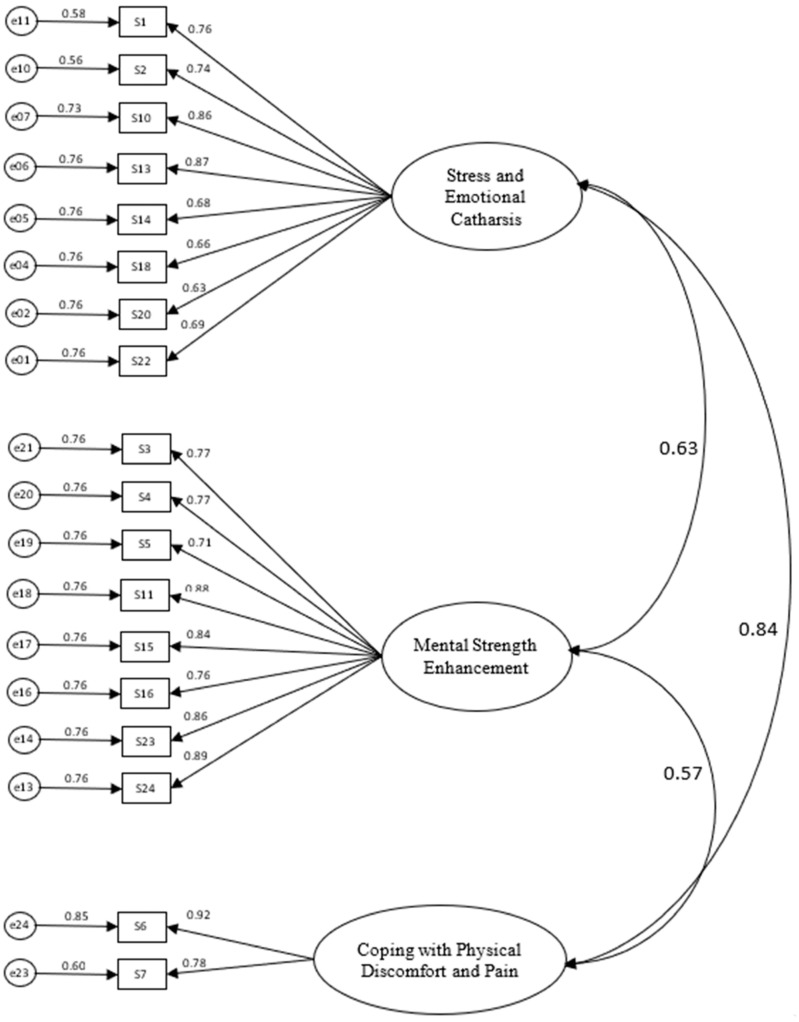
A 3-dimension CFA solution (factor loadings) for the Reasons for Swearing in Sport and Exercise Questionnaire *(RSSEQ)*.

The model’s fit coefficients are presented in Table 3. Model 1, which included all 23 items, resulted in suboptimal fit indices: *χ*^2^(249) = 804.70, *p* < 0.001, *χ*^2^/*df* = 3.23, *CMIN/df* = 3.23, *CFI* = 0.85, *TLI* = 0.83, *RMSEA* = 0.11, and *SRMR* = 0.08. These fit indices indicated a need for modifications to improve the model’s fit to the data. Items with high residual variances (>1) and weak factor loadings were identified and removed. Specifically, items 8, 9, and 19 were eliminated from the Stress and Emotional Catharsis factor; item 17 was removed from the Mental Strength Enhancement factor; and item 12 was removed from the Coping with Physical Pain and Discomfort factor. The refined model (Model 2) showed substantial improvements in its fit indices: *χ*^2^(131) = 235.29, *p* < 0.001, *χ*^2^/*df* = 1.79, *CFI* = 0.92, *TLI* = 0.95, *RMSEA* = 0.06, and *SRMR* = 0.05. All fit indices for Model 2 met or exceeded the recommended thresholds, indicating a strong fit between the refined measurement model and the observed data. Reliability analyses demonstrated high internal consistency across factors, with CA values of 0.93 for Factor 1, 0.94 for Factor 2, and 0.83 for Factor 3. Figure 1 illustrates the final model’s factors and items’ loadings. Table 4 presents the values for CA, CR, and AVE. Factor 1 resulted in CA = 0.93, CR = 0.93, and AVE = 0.61. Factor 2 showed a CA = 0.94, CR = 0.94, and AVE = 0.64. Factor 3 yielded a CA = 0.83, CR = 0.84, and AVE = 0.73. These values indicate good to excellent reliability and validity, supporting the robustness of the measurement model. Discriminant validity was assessed using the Fornell–Larcker criterion (Table 5) and the Heterotrait–Monotrait ratio (HTMT) (Table 6). As shown in Table 4, the square roots of the AVE values (bold diagonal) exceeded the inter-factor correlations, confirming sufficient discriminant validity (Fornell & Larcker, 1981). Additionally, all HTMT values were below the conservative threshold of 0.85, affirming adequate discriminant validity (Henseler et al., 2015). These findings suggest that each dimension is sufficiently distinct, ensuring the model measures unique theoretical concepts.

### 3.3. Validity Procedures

Given the lack of validated measures that can be used to assess swearing, aggression and anger were chosen to construct-validate the RSSEQ. Research findings consistently show that swearing functions as a means of emotional expression, particularly for negative emotions like anger, frustration, and aggression (Jay & Janschewitz, 2008; Popuşoi et al., 2018; Stapleton, 2003; Rassin & Muris, 2005). Although ST constructs, such as the ‘Big Five’ personality traits, were considered, aggression and anger were emphasized due to their stronger empirical connections to the functional role of swearing. Additionally, gender, professional level, and sport type were analyzed to capture demographic and contextual variations in swearing behavior. The inclusion of exercisers, athletes, and coaches allowed for a comprehensive examination across different levels of sport involvement, fostering a nuanced understanding of how swearing functions within various roles and competitive contexts.

### 3.4. Construct Validity

A multivariate analysis of variance (MANOVA) was conducted to test the effects of gender (i.e., males vs. females), professional status (i.e., competitive vs. recreational), professional role (i.e., athlete vs. coach), and sport type (i.e., individual vs. team) on the three swearing-motivation dimensions. Gender resulted in a significant but small multivariate effect: *Wilks’ λ* = 0.927, *F*(3, 326) = 6.38, *p* < 0.001, *ηp*^2^ = 0.073. Follow-up ANOVAs indicated the non-significant effect of gender on Stress and Emotional Catharsis, *F*(1, 328) = 0.86, *p* = 0.35, *ηp*^2^ = 0.003, suggesting that males and females reported similar motivations for swearing as a form of emotional release. However, significant gender effects were identified for the other two factors, with Mental Strength Enhancement exhibiting a moderate effect, *F*(1, 328) = 18.67, *p* < 0.001, *ηp*^2^ = 0.054, and Coping with Physical Discomfort and Pain revealing a small-to-moderate effect, *F*(1, 328) = 7.72, *p* = 0.006, *ηp*^2^ = 0.023. These findings imply that while gender does not influence the use of swearing for stress relief, it does affect the motivation to swear for the purpose of enhancing mental strength and coping with physical pain and discomfort, with males showing higher usage than females. Professional level resulted in a small multivariate effect: *Wilks’ λ* = 0.970, *F*(3, 330) = 3.44, *p* = 0.02, *pη*^2^ = 0.030. Follow-up ANOVAs showed non-significant effects of professional level on Stress and Emotional Catharsis, *F*(1, 332) = 1.66, *p* = 0.198, *ηp*^2^ = 0.005; Mental Strength Enhancement, *F*(1, 332) = 0.82, *p* = 0.366, *ηp*^2^ = 0.002; and Coping with Physical Discomfort and Pain, *F*(1, 332) = 1.39, *p* = 0.24, *ηp*^2^ = 0.004; these results indicated no meaningful differences in swearing motivations between competitive and recreational athletes. Sport type resulted in a non-significant multivariate effect: *Wilks’ λ* = 0.95, *F*(3, 160) = 2.51, *p* = 0.061, *ηp*^2^ = 0.045. Professional role (i.e., athlete vs. coach) yielded a statistically significant multivariate effect, *Wilks’ λ* = 0.963, *F*(3, 247) = 3.14, *p* = 0.026, *ηp^2^* = 0.037, indicating a small overall effect. Follow-up ANOVAs revealed that professional role significantly influenced Stress and Emotional Catharsis, *F*(1, 249) = 7.57, *p* = 0.006, *ηp^2^* = 0.029, with athletes reporting higher usage than coaches. No significant differences were found for Mental Strength Enhancement (MSE), *F*(1, 249) = 2.64, *p* = 0.106, *ηp^2^* = 0.010; or for Coping with Discomfort and Pain (CDP), *F*(1, 249) = 0.08, *p* = 0.772, *ηp^2^* < 0.001. In summary, gender resulted in a small-to-moderate effect on swearing motivation, particularly for Mental Strength Enhancement and Coping with Physical Discomfort and Pain. Professional role revealed a small but significant effect, specifically, for Stress and Emotional Catharsis, with athletes reporting higher usage than coaches. However, sport type did not yield any meaningful differences across the three swearing-motivation factors.

### 3.5. Convergent Validity

The convergent validity of the RSSEQ was assessed by inspecting its correlations with the BAQ and CAAS questionnaires (see Table 7). Significant positive moderate correlations were evident between the RSSEQ and both the BAQ overall score (*r* = 0.42, *p* < 0.001) and the CAAS overall score (*r* = 0.44, *p* < 0.001), supporting the theoretical alignment of the RSSEQ with these established psychological measures. With reference to the two CAAS factors, Anger was significantly and positively correlated with Stress and Emotional Catharsis (*r* = 0.45, *p* < 0.01) and with Coping with Physical Discomfort and Pain (*r* = 0.25, *p* < 0.01). In contrast, its correlation with Mental Strength Enhancement was not statistically significant (*r* = 0.13, *p* = 0.09). Aggressiveness was significantly and positively correlated with Stress and Emotional Catharsis (*r* = 0.26, *p* < 0.01), Mental Strength Enhancement (*r* = 0.33, *p* < 0.01), and Coping with Physical Discomfort and Pain (*r* = 0.29, *p* < 0.01). The correlations of the four BAQ factors were as follows: Physical Aggression was significantly and positively correlated with Stress and Emotional Catharsis (*r* = 0.33, *p* < 0.01), Mental Strength Enhancement (*r* = 0.31, *p* < 0.01), and Coping with Physical Discomfort and Pain (*r* = 0.38, *p* < 0.01). Anger was also significantly correlated with Stress and Emotional Catharsis (*r* = 0.21, *p* < 0.01), Mental Strength Enhancement (*r* = 0.21, *p* < 0.01), and Coping with Physical Discomfort and Pain (*r* = 0.19, *p* < 0.05). Verbal Aggression failed to significantly correlate with Stress and Emotional Catharsis (*r* = 0.15, *p* = 0.056), Mental Strength Enhancement (*r* = 0.13, *p* = 0.08), or Coping with Physical Discomfort and Pain (*r* = 0.14, *p* = 0.07). Hostility was positively associated with Stress and Emotional Catharsis (*r* = 0.30, *p* < 0.01), Mental Strength Enhancement (*r* = 0.16, *p* < 0.05), and Coping with Physical Discomfort and Pain (*r* = 0.26, *p* < 0.01).

### 3.6. Temporal Stability

A temporal stability analysis was performed to evaluate the consistency of participants’ responses over time. Responses were collected from 92 participants who underwent EFA at two points separated by a three-month interval. Spearman correlations were conducted for each item, with values ranging from 0.58 to 0.90; all *p* < 0.001, indicating strong temporal stability. The full item-level results are presented in Table 8.

## 4. Discussion

This study aimed to develop a valid and reliable questionnaire that could be used to assess the motivation for swearing, as an integral aspect of ST, among athletes, coaches, and recreational exercisers. To the best of our knowledge, this study is the first to provide empirical data and insights into the reasons sport and exercise participants swear during ST, offering a foundation for future research on this topic. Although swear words have distinct psychological effects that other forms of language miss (Stapleton et al., 2022), swearing has long been overlooked in academic research, often dismissed as “trivial, distasteful, or otherwise unworthy of serious scholarship” (Stapleton & Beers Fägersten, 2023, p. 148). However, recent studies have illuminated various positive effects of swearing, including enhanced pain-tolerance (Hay et al., 2024) and physical performance (Washmuth et al., 2024), and a state of increased self-confidence (Stephens et al., 2023). In advancing this growing field, a key contribution of this study was the development of a psychometrically sound questionnaire that systematically examines the motivations behind the use of swear words, as a function of ST, among sport and exercise participants.

The RSSEQ resulted in a well-structured three-factor model encompassing 18 items, with each factor capturing a distinct motivational dimension for swearing: Stress and Emotional Catharsis (eight items), Mental Strength Enhancement (eight items), and Coping with Physical Discomfort and Pain (two items). The final model demonstrated a good overall fit, with all key indices meeting or exceeding recommended thresholds. The comparative fit indices indicated an adequate-to-strong model fit. Although some indices failed to reach the threshold for an excellent fit, the results confirm that the refined questionnaire provides a robust and reliable measurement framework, effectively capturing the intended dimensions and ensuring its suitability for research in sport and exercise psychology.

The first factor, Stress and Emotional Catharsis, captures the tendency to use swearing to release tension, frustration, or emotional distress during sports or exercise. This dimension reflects the notion that swearing serves as a psychological release valve (Vingerhoets et al., 2013), allowing individuals to express intense emotions and relieve mental strain in high-pressure or physically demanding situations (Jay & Janschewitz, 2008). This is consistent with prior research, since swearing has been recognized as an effective coping mechanism for emotional regulation, providing a swift and instinctive response to stress (Husain et al., 2023; Stephens & Zile, 2017). In sports contexts, where athletes and coaches frequently face performance-related pressure, swearing may provide an immediate and accessible tool for managing negative emotions, maintaining composure, and refocusing attention. For example, a tennis player who misses a crucial shot might swear under their breath to quickly vent frustration and mentally reset for the next point. From a practical standpoint, acknowledging this function of swearing can help sport psychologists, coaches, and practitioners understand its role in emotional self-regulation and potentially incorporate swear words into mental skills training to enhance emotional resilience and stress management in competitive and emotionally challenging settings.

The second factor, Mental Strength Enhancement, represents swearing as a tool for boosting confidence, determination, and resilience. Unlike emotional release, it emphasizes psychological empowerment, helping athletes to overcome anxiety, stay focused on the task, and sustain effort in challenging moments. It can also be considered a form of motivational ST, as it involves the intentional use of language to psychologically prime oneself for stressful and challenging moments (Hardy et al., 2018; Theodorakis et al., 2000). Future research must target the potential differences between traditional motivational ST (e.g., ‘you can do this!’) and swearing-based ST (e.g., ‘you’re fucking beating this guy!’) as to their effects on physical performance and psychological constructs such as self-efficacy, grit, and mental toughness. From an applied perspective, acknowledging that swearing can act as a motivational tool for certain individuals may assist practitioners in understanding athletes’ behaviors and developing tailored psychological strategies, when culturally and ethically suitable. However, it is not recommended to encourage practitioners to use strong language if they are uncomfortable with it. Instead, assessing their willingness to incorporate such language and ensuring that it aligns with their personal preferences is advisable.

The third factor, Coping with Physical Discomfort and Pain, encompasses swearing as a mechanism used for enduring physical exertion, fatigue, and pain during sport and exercise. In this context, swearing is a pain modulation strategy, allowing individuals to temporarily override discomfort and maintain effort during physically demanding tasks. Research findings support this effect, emphasizing the utility of swearing as a valuable psychological tool for athletes and exercisers, one used to effectively deal with unpleasant physical sensations (Hay et al., 2024). For example, during an intense training session, a basketball player performing a grueling drill might mutter a swear word under their breath to ‘push through’ the pain and maintain effort. This strategy is not limited to sport and exercise; it can also be applied in other contexts, such as physiotherapy, a field in which certain activities or rehabilitation exercises may induce pain (Washmuth & Stephens, 2022). In such settings, swearing can serve as a temporary coping mechanism to help patients manage discomfort and adhere to treatment plans. By recognizing this aspect of swearing, sports and exercise practitioners, along with healthcare professionals, can better understand its role in assisting individuals during physically painful moments.

Analysis of gender differences in swearing motivation revealed that males were more likely to swear for mental strength enhancement and coping with physical discomfort and pain, while no significant difference was observed for stress or emotional catharsis. This indicates that both males and females are similarly motivated to swear to relieve stress and tension during frustrating situations, such as missing an important shot or being substituted for against their will, while males tend more often to use swearing as a psychological priming tool, compared to females. In light of that, some research shows that male athletes report lower states of anxiety during competition, compared to female athletes (Dias et al., 2010), suggesting that male athletes may naturally adopt strategies that reinforce higher mental toughness (Nicholls et al., 2008; Sheard et al., 2009). Given that swearing is believed to provide psychological benefits which extend beyond conventional language (Stapleton et al., 2022), it is possible that male athletes engage in swearing—consciously or unconsciously (Jay, 2009)—to optimize their psychological readiness for competition. For example, Stephens et al. (2023) found that swearing before a physical task enhanced the state of self-confidence, a key component of mental toughness (Clough et al., 2002; Jones et al., 2002, 2007). Moreover, swearing before or during challenging moments may promote a challenge state rather than a threat state, enhancing emotional preparedness and adaptive arousal. Thus, in certain contexts, swearing may support psychological readiness, even within populations less likely to engage in such behavior. However, this hypothesis requires further investigation. Specifically, we did not assess factors which might prevent individuals from using swear words, and are thus unable to determine with certainty whether females swear less due to social norms. Future studies must explore potential barriers that discourage female athletes, coaches, and recreational exercisers from swearing, as no research has directly examined this question within an athletic population. Furthermore, more research must target the questions of whether swearing actively contributes to pre-competition self-confidence and emotional regulation, and when athletes swear most frequently—whether as part of a pre-performance routine to prepare mentally or during the competition itself to sustain effort and manage adversity. Recognizing these patterns will provide deeper insights into the strategic use of swearing in high-performance settings.

We also found that professional levels (e.g., competitive athletes and coaches vs. recreational athletes) were not associated with significant effects on swearing motivation, indicating that both professional and recreational athletes share the same motivation for swearing. This finding suggests that using swearing as a psychological and performance-related tool is not exclusive to high-level competition but rather a broader phenomenon across different levels of sport and exercise participation. One possible explanation is that swearing is a deeply ingrained and instinctive verbal response to emotional, cognitive, and physical stressors (Van Lancker & Cummings, 1999), making it a natural reaction regardless of one’s level of competitiveness or professional involvement in sport. Therefore, the fact that both cohorts exhibit similar swearing patterns may reflect its universal function in managing effort, sustaining motivation, and regulating emotions in physically and mentally demanding situations. In practical terms, these findings suggest that swearing can be relevant as a psychological tool across different levels of sport participation.

Sport type (individual vs. team sports) failed to significantly influence swearing motivation. This suggests that athletes and coaches in both individual and team sports use swearing for similar reasons, across all three factors. We speculate that individual and team sport athletes experience similar psychological and physical demands, such as performance pressure, pain tolerance, and motivation. Whether competing alone or within a team, athletes still face moments of frustration, fatigue, and high-intensity effort, moments in which swearing may provide a spontaneous and instinctive way to regulate emotions and maintain performance.

Finally, athletes reported a significantly greater use of swearing for emotional release than did coaches, likely because of the immediate physical and emotional pressures athletes encounter during performance. In contrast, coaches may utilize more controlled verbal techniques that align with their leadership responsibilities. Nonetheless, no significant differences were found regarding mental strength enhancement, indicating that both athletes and coaches consider intense language a legitimate motivational tool. However, one must consider that the sample consisted of more athletes than coaches, which could have affected the outcomes and limited the generalizability of the findings. More research using more balanced samples of athletes and coaches is required to further explore potential differences in swearing motivations related to these roles.

### Limitations

This study has several limitations which must be considered when interpreting the findings. First, the sample consisted exclusively of Israeli participants, limiting the generalizability of the results to other cultural contexts. Since language, social norms, and attitudes toward swearing vary across countries and cultures, future research must validate the questionnaire in diverse populations. Second, swearing is a socially and culturally sensitive topic (Jay & Janschewitz, 2008), and participants may have underreported or misrepresented their motivations due to social desirability bias or personal discomfort, particularly those from religious or conservative backgrounds. Third, as the study relied on self-reporting measures, participants may have had difficulty accurately recalling the reasons for their swearing during sport, leading to potential recall bias. Additionally, since the sample included both competitive and recreational athletes, differences in swearing motivation across various levels of competition were not examined in detail (e.g., elite vs. regional, youth vs. adults). Similarly, while no significant differences were found between individual and team sport athletes, the study did not explore variations across specific sport types (e.g., contact vs. non-contact sports, endurance vs. short-duration sports) that might possibly influence swearing behaviors.

## 5. Conclusions

This study developed and validated the RSSEQ, a novel questionnaire designed to examine the motivation for swearing, as a function of ST, among athletes, coaches, and recreational exercisers. The findings identified three distinct motivational dimensions: stress and emotional catharsis, mental strength enhancement, and coping with physical discomfort and pain. By establishing a reliable framework for measuring swearing motivation, this study lays the groundwork for further research into the psychological and performance-related functions of swearing in sport and exercise contexts. We encourage future research to go beyond the consideration of swearing as a taboo or trivial topic and instead examine its potential benefits as a form of emotionally charged language that may enhance mental regulation, resilience, and physical performance in competitive and demanding situations.

## Figures and Tables

**Table 1:** List of initial 26 reasons for swearing as a form of self-talk in sport and exercise contexts.

Item	Reason for Swearing
S1	To alleviate frustration in challenging moments
S2	To ‘blow off steam’ in stressful or intense situations
S3	To boost your self-confidence
S4	To maintain motivation in difficult times
S5	To focus on the task at hand
S6	To cope with physical pain or discomfort
S7	To distract from physical pain caused by injury/hit
S8	To delay or redirect your aggression
S9	To emphasize the intensity of the situation to teammates or coaches
S10	To release nerves and tension
S11	To boost your motivation for better performance when you need mental reinforcement
S12	To psychologically reduce the impact of physical pain during your sports performance
S13	To vent frustration instead of keeping it inside
S14	To feel less bad about mistakes by verbally expressing disappointment
S15	To mentally prepare for the next move or strategy
S16	To amplify feelings of joy or success in positive moments
S17	To tap into a sense of aggression that supports optimal performance
S18	To cope with disappointment or nerves
S19	To alleviate frustration in challenging moments
S20	To manage feelings of frustration when things don’t go as planned
S21	To channel anger into improved performance
S22	To quickly release anger
S23	To express positive emotions and celebrate successes
S24	To cope with pressure
S25	To clear out negative emotions
S26	To regain mental clarity

Note. These 26 items reflect the reasons participants reported for using swearing as a form of self-talk during sport and exercise activities. The items consisted of the responses of 15 participants in the initial qualitative phase of the study, in combination with existing findings from the literature.

**Table 2:** EEFA 3-factor pattern (dimension) solution following oblimin rotation.

Swearing Items	Stress and Emotional Catharsis	Mental Strength Enhancement	Coping with Physical Discomfort and Pain
S1—Alleviate feelings of frustration during challenging moments.	0.70		
S2—“Let off steam” in stressful or intense situations.	0.68		
S8—Delay or redirect your aggression.	0.50		
S9—Emphasize the intensity of the situation to teammates or coaches.	0.55		
S10—Release nerves and tension.	0.84		
S13—Vent frustration instead of keeping it bottled up.	0.87		
S14—Feel less bad about mistakes by verbally expressing disappointment.	0.64		
S18—Cope with disappointment or frustration.	0.81		
S19—Express anger in frustrating moments.	1.00		
S20—Manage feelings of frustration when things don’t go as planned.	0.79		
S22—Quickly release anger.	0.69		
S3—Boost your self-confidence.		0.71	
S4—Maintain motivation during difficult times.		0.75	
S5—Express positive emotions and celebrate successes.		0.65	
S11—Increase motivation for better performance when in need of a mental boost.		0.73	
S15—Mentally prepare for the next move or strategy.		0.81	
S16—Enhance feelings of joy or success during positive moments.		0.72	
S17—Enter an aggressive mindset that facilitates peak performance.		0.59	
S23—Stay focused on the task.		0.81	
S24—Handle pressure.		0.46	
S6—Cope with pain or physical discomfort.			0.63
S7—Distract yourself from physical pain caused by injury or impact.			0.84
S12—Psychologically reduce the impact of physical pain during athletic performance.			0.65

**Table 3:** Goodness-of-fit statistics for Model 1 (expanded number of items) and Model 2 (final number of items).

	*χ* ^2^	*df*	CMIN/DF	CFI	TLI	RMSEA	SRMR
Model 1	804.698	249	3.232	0.845	0.828	0.112	0.0827
Model 2	235.291	131	1.792	0.916	0.949	0.067	0.0514

**Table 4:** Measurement properties of reflective dimensions.

Constructs	Items	α	CR	AVE
1. Dimension 1	8	0.93	0.93	0.61
2. Dimension 2	9	0.94	0.94	0.64
3. Dimension 3	2	0.83	0.84	0.73

**Table 5:** Discriminant validity of the measures (Fornell–Larcker criteria).

Variable	1	2	3
1. Dimension 1	0.78		
2. Dimension 2	0.63 ***	0.80	
3. Dimension 3	0.84 ***	0.57 ***	**0.85**

Note. *** *p* < 0.001, Bold diagonal values indicate square root of AVE.

**Table 6:** Heterotrait–Monotrait criteria (HTMT) for discriminant validity.

Dimension	1	2	3
1. Dimension 1	-		
2. Dimension 2	0.66	-	
3. Dimension 3	0.84	0.59	-

**Table 7:** Pearson product-moment correlations between the Reasons for Swearing in Sport and Exercise Questionnaire dimensions (RSSEQ), the Brief Aggression Questionnaire (BAQ) and its dimensions, and the Competitive Aggressiveness and Anger Scale (CAAS) and its dimensions.

	(1)	(2)	(3)	(4)	(5)	(6)	(7)	(8)	(9)	(10)	(11)	(12)
(1) SEC	1											
(2) MSE	0.55 **											
(3) CPDP	0.66 **	0.52 **										
(4) Physical Aggression—BAQ	0.32 **	0.30 **	0.38 **									
(5) Anger—BAQ	0.20 **	0.21 **	0.19 **	0.18 **								
(6) Verbal Aggression—BAQ	0.14	0.13	0.14	0.23 **	0.24 **							
(7) Hostility—BAQ	0.30 **	0.15 *	0.26 **	0.13	0.18 *	0.11						
(8) RSSEQ	0.87 **	0.87 **	0.74 **	0.38 **	0.24 **	0.16 *	0.27 **					
(9) BAQ	0.39 **	0.31 **	0.39 **	0.62 **	0.52 **	0.77 **	0.68 **	0.62 **				
(10) CAAS	0.48 **	0.28 **	0.33 **	0.41 **	0.21 **	0.28 **	0.28 **	0.44 **	0.49 **			
(11) Anger—CAAS	0.45 **	0.13	0.24 **	0.16 *	0.18 *	0.22 *	0.34 **	0.33 **	0.38 **	0.83 **		
(12) Aggressiveness—CAAS	0.26 **	0.32 **	0.29 **	0.53 **	0.14	0.22 **	0.05	0.33 **	0.38 **	0.69 **	0.18 *	

Note: * *p* < 0.05, ** *p* < 0.01. SEC = Stress and Emotional Catharsis; MSE = Mental Strength Enhancement; CPDP = Coping with Discomfort and Pain; CAAS = The Competitive Aggressiveness and Anger Scale; BAQ = Brief Aggression Questionnaire.

**Table 8:** Temporal Stability for individual items over a three-month interval (N = 92).

Construct	Item	Spearman’s ρ	*p*-Value
SEC	S1	0.76	<0.001
	S2	0.70	<0.001
	S3	0.58	<0.001
	S10	0.78	<0.001
	S13	0.81	<0.001
	S14	0.68	<0.001
	S20	0.73	<0.001
	S27	0.75	<0.001
MSE	S11	0.90	<0.001
	S4	0.77	<0.001
	S5	0.71	<0.001
	S15	0.83	<0.001
	S16	0.80	<0.001
	S18	0.72	<0.001
	S23	0.76	<0.001
	S24	0.74	<0.001
CPDP	S6	0.85	<0.001
	S7	0.80	<0.001

Note. Test–retest reliability was assessed using Spearman’s rank-order correlation across a three-month interval. All correlations were statistically significant at *p* < 0.001. SEC = Stress and Emotional Catharsis; MSE = Mental Strength Enhancement; CDP = Coping with Physical Discomfort and Pain.

## Data Availability

The data are available from the corresponding author upon reasonable request, in order to ensure appropriate use and interpretation of the dataset.

## References

[B1-behavsci-15-00593] Adebiyi J. Y., Kyrillou C., Martinez E., Patel A., Payne E., Chen L., Suksom D., Tanaka H. (2025). Does swearing improve exercise performance and pain tolerance?: Cultural perspective. International Journal of Exercise Science: Conference Proceedings.

[B2-behavsci-15-00593] Ballmann C. G., Jiannine L., Washmuth N. B. (2024). Exploring the effects of swearing on aerobic endurance: A preliminary inv estigation. Journal of Exercise Physiology Online.

[B3-behavsci-15-00593] Beck V., Brooks J. L., Stephens R. (2025). The effect of swearing on error-related negativity as an indicator for state disinhibition. Quarterly Journal of Experimental Psychology.

[B4-behavsci-15-00593] Beers Fägersten K. (2012). Who’s swearing now? The social aspects of conversational swearing.

[B5-behavsci-15-00593] Buss A. H., Perry M. (1992). The aggression questionnaire. Journal of Personality and Social Psychology.

[B6-behavsci-15-00593] Clough P. J., Earle K., Sewell D., Cockerill I. (2002). Mental toughness: The concept and its measurement. Solutions in sport psychology.

[B7-behavsci-15-00593] Dali M. S. B., Parnabas V. A. (2018). The effects of self-talk on free throw performance and the level of anxiety among male novice basketball players. Malaysian Journal of Movement, Health & Exercise.

[B8-behavsci-15-00593] De Vellis R. F. (2003). Scale development: Theory and applications.

[B9-behavsci-15-00593] Dewaele J. M. (2004). The emotional force of swearwords and taboo words in the speech of multilinguals. Journal of Multilingual and Multicultural Development.

[B10-behavsci-15-00593] Dias C., Cruz J. F., Fonseca A. M. (2010). Coping strategies, multidimensional competitive anxiety and cognitive threat appraisal: Differences across sex, age and type of sport. Serbian Journal of Sports Sciences.

[B11-behavsci-15-00593] Fabrigar L. R., Wegener D. T., MacCallum R. C., Strahan E. J. (1999). Evaluating the use of exploratory factor analysis in psychological research. Psychological Methods.

[B12-behavsci-15-00593] Fornell C., Larcker D. F. (1981). Evaluating structural equation models with unobservable variables and measurement error. Journal of Marketing Research.

[B13-behavsci-15-00593] Galanis E., Hatzigeorgiadis A., Charachousi F., Latinjak A. T., Comoutos N., Theodorakis Y. (2022). Strategic self-talk assists basketball free throw performance under conditions of physical exertion. Frontiers in Sports and Active Living.

[B14-behavsci-15-00593] Hardy J. (2006). Speaking clearly: A critical review of the self-talk literature. Psychology of Sport and Exercise.

[B15-behavsci-15-00593] Hardy J., Comoutos N., Hatzigeorgiadis A. (2018). Reflections on the maturing research literature of self-talk in sport: Contextua lizing the special issue. The Sport Psychologist.

[B16-behavsci-15-00593] Hardy J., Oliver E., Tod D. (2008). A framework for the study and application of self-talk within sport. Advances in applied sport psychology.

[B17-behavsci-15-00593] Hatzigeorgiadis A., Galanis E. (2017). Self-talk effectiveness and attention. Current Opinion in Psychology.

[B18-behavsci-15-00593] Hatzigeorgiadis A., Zourbanos N., Galanis E., Theodorakis Y. (2011). Self-talk and sports performance: A meta-analysis. Perspectives on Psychological Science.

[B19-behavsci-15-00593] Hay C. M., Sills J. L., Shoemake J. M., Ballmann C. G., Stephens R., Washmuth N. B. (2024). F@# $ pain! A mini-review of the hypoalgesic effects of swearing. Frontiers in Psychology.

[B20-behavsci-15-00593] Henseler J., Ringle C. M., Sarstedt M. (2015). A new criterion for assessing discriminant validity in variance-based structural equation modeling. Journal of the Academy of Marketing Science.

[B21-behavsci-15-00593] Hong X., Liao Y., Shi Y., Qi C., Zhao M., Van Raalte J. L. (2020). An empirical test of the self-talk dissonance hypothesis: The effects of self-talk overtness and personality on performance. The Sport Psychologist.

[B22-behavsci-15-00593] Hufton J. R., Vella S. A., Goddard S. G., Schweickle M. J. (2024). How do athletes perform well under pressure? A meta-study. International Review of Sport and Exercise Psychology.

[B23-behavsci-15-00593] Husain W., Wasif S., Fatima I. (2023). Profanity as a self-defense mechanism and an outlet for emotional catharsis in stress, anxiety, and depression. Depression Research and Treatment.

[B24-behavsci-15-00593] Jay T. (2009). The utility and ubiquity of taboo words. Perspectives on Psychological Science.

[B25-behavsci-15-00593] Jay T., Allan K. (2018). The psychology of expressing and interpreting linguistic taboos. The Oxford handbook of taboo words and language.

[B26-behavsci-15-00593] Jay T., Janschewitz K. (2008). The pragmatics of swearing. Journal of Politeness Research.

[B27-behavsci-15-00593] Jones G., Hanton S., Connaughton D. (2002). What is this thing called mental toughness? An investigation of elite sport performers. Journal of Applied Sport Psychology.

[B28-behavsci-15-00593] Jones G., Hanton S., Connaughton D. (2007). A framework of mental toughness in the world’s best performers. The Sport Psychologist.

[B29-behavsci-15-00593] Kahneman D. (2011). Thinking, fast and slow.

[B30-behavsci-15-00593] Kline R. B. (2015). Principles and practice of structural equation modeling.

[B31-behavsci-15-00593] Kross E., Bruehlman-Senecal E., Park J., Burson A., Dougherty A., Shablack H., Bremner R., Moser J., Ayduk O. (2014). Self-talk as a regulatory mechanism: How you do it matters. Journal of Personality and Social Psychology.

[B32-behavsci-15-00593] Lakens D. (2022). Sample size justification. Collabra: Psychology.

[B33-behavsci-15-00593] Latinjak A. T., Morin A., Brinthaupt T. M., Hardy J., Hatzigeorgiadis A., Kendall P. C., Neck C., Oliver E., Puchalska-Wasyl M. M., Tovares A. V., Winsler A. (2023). Self-talk: An interdisciplinary review and transdisciplinary model. Review of General Psychology.

[B34-behavsci-15-00593] Latinjak A. T., Zourbanos N., López-Ros V., Hatzigeorgiadis A. (2014). Goal-directed and undirected self-talk: Exploring a new perspective for the study of athletes’ self-talk. Psychology of Sport and Exercise.

[B35-behavsci-15-00593] Lindquist K. A. (2017). The role of language in emotion: Existing evidence and future directions. Current Opinion in Psychology.

[B36-behavsci-15-00593] Lindquist K. A., MacCormack J. K., Shablack H. (2015). The role of language in emotion: Predictions from psychological costructionism. Frontiers in Psychology.

[B37-behavsci-15-00593] Lupyan G., Clark A. (2015). Words and the world: Predictive coding and the language-perception-cognition interface. Current Directions in Psychological Science.

[B38-behavsci-15-00593] Maxwell J. P., Moores E. (2007). The development of a short scale measuring aggressiveness and anger in competitive athletes. Psychology of Sport and Exercise.

[B39-behavsci-15-00593] Montagu A. (2001). The anatomy of swearing.

[B40-behavsci-15-00593] Nedergaard J., Christensen M. S., Wallentin M. (2021). Valence, form, and content of self-talk predict sport type and level of performance. Consciousness and Cognition.

[B41-behavsci-15-00593] Nicholls A. R., Polman R. C., Levy A. R., Backhouse S. H. (2008). Mental toughness, optimism, pessimism, and coping among athletes. Personality and Individual Differences.

[B42-behavsci-15-00593] Nunally J. C. (1978). Psychometric theory.

[B43-behavsci-15-00593] OpenAI (2025). ChatGPT (March 7 version).

[B44-behavsci-15-00593] Oppenheim G. M., Dell G. S. (2010). Motor movement matters: The flexible abstractness of inner speech. Memory & Cognition.

[B45-behavsci-15-00593] Pinker S. (2007). The stuff of thought: Language as a window into human nature.

[B46-behavsci-15-00593] Popuşoi S. A., Havârneanu G. M., Havârneanu C. E. (2018). “Get the f#∗ k out of my way!” Exploring the cathartic effect of swear words in coping with driving anger. Transportation Research Part F: Traffic Psychology and Behaviour.

[B47-behavsci-15-00593] Rassin E., Muris P. (2005). Why do women swear? An exploration of reasons for and perceived efficacy of swearing in Dutch female students. Personality and Individual Differences.

[B48-behavsci-15-00593] Rhodes R. E., Kates A. (2015). Can the affective response to exercise predict future motives and physical activity behavior? A systematic review of published evidence. Annals of Behavioral Medicine.

[B49-behavsci-15-00593] Rosenberg P., Sikström S., Garcia D. (2017). The a(b)(c) of taboo words in natural language: The relationship between taboo words’ intensity and frequency. Journal of Language and Social Psychology.

[B50-behavsci-15-00593] Sheard M., Golby J., Van Wersch A. (2009). Progress toward construct validation of the Sports Mental Toughness Questionnaire (SMTQ). European Journal of Psychological Assessment.

[B51-behavsci-15-00593] Stapleton K. (2003). Gender and swearing: A community practice. Women and Language.

[B52-behavsci-15-00593] Stapleton K., Locher M., Graham S. L. (2010). Swearing. Interpersonal pragmatics.

[B53-behavsci-15-00593] Stapleton K., Beers Fägersten K. (2023). Editorial: Swearing and interpersonal pragmatics. Journal of Pragmatics.

[B54-behavsci-15-00593] Stapleton K., Beers Fägersten K., Stephens R., Loveday C. (2022). The power of swearing: What we know and what we don’t. Lingua.

[B55-behavsci-15-00593] Stephens R., Dowber H., Barrie A., Almeida S., Atkins K. (2023). Effect of swearing on strength: Disinhibition as a potential mediator. Quarterly Journal of Experimental Psychology.

[B56-behavsci-15-00593] Stephens R., Spierer D. K., Katehis E. (2018). Effect of swearing on strength and power performance. Psychology of Sport and Exercise.

[B57-behavsci-15-00593] Stephens R., Zile A. (2017). Does emotional arousal influence swearing fluency?. Journal of Psycholinguistic Research.

[B58-behavsci-15-00593] Stone T. E., Hazelton M. (2008). An overview of swearing and its impact on mental health nursing practice. International Journal of Mental Health Nursing.

[B59-behavsci-15-00593] Theodorakis Y., Weinberg R., Natsis P., Douma I., Kazakas P. (2000). The effects of motivational versus instructional self-talk on improving motor performance. The Sport Psychologist.

[B60-behavsci-15-00593] Tod D., Edwards C., McGuigan M., Lovell G. (2015). A systematic review of the effect of cognitive strategies on strength performance. Sports Medicine.

[B61-behavsci-15-00593] Tod D., Hardy J., Oliver E. (2011). Effects of self-talk: A systematic review. Journal of Sport and Exercise Psychology.

[B62-behavsci-15-00593] Van Lancker D., Cummings J. L. (1999). Expletives: Neurolinguistic and neurobehavioral perspectives on swearing. Brain Research Reviews.

[B63-behavsci-15-00593] Van Raalte J. L., Brewer B. W., Rivera P. M., Petitpas A. J. (1994). The relationship between observable self-talk and competitive junior tennis players’ match performances. Journal of Sport and Exercise Psychology.

[B64-behavsci-15-00593] Van Raalte J. L., Vincent A., Tod D., Hodge K., Krane V. (2023). Self-talk. Routledge handbook of applied sport psychology.

[B65-behavsci-15-00593] Van Raalte J. L., Vincent A., Brewer B. W. (2016). Self-talk: Review and sport-specific model. Psychology of Sport and Exercise.

[B66-behavsci-15-00593] Vingerhoets A. J., Bylsma L. M., De Vlam C. (2013). Swearing: A biopsychosocial perspective. Psychological Topics.

[B67-behavsci-15-00593] Wallace P. J., McKinlay B. J., Coletta N. A., Vlaar J. I., Taber M. J., Wilson P. M., Cheung S. S. (2017). Effects of motivational self-talk on endurance and cognitive performance in the heat. Medicine and Science in Sports and Exercise.

[B68-behavsci-15-00593] Walter N., Nikoleizig L., Alfermann D. (2019). Effects of self-talk training on competitive anxiety, self-efficacy, volitional skills, and performance: An intervention study with junior sub-elite athletes. Sports.

[B69-behavsci-15-00593] Washmuth N. B., Stephens R. (2022). Frankly, we do give a damn: Improving patient outcomes with swearing. Archives of Physiotherapy.

[B70-behavsci-15-00593] Washmuth N. B., Stephens R., Ballmann C. G. (2024). Effect of swearing on physical performance: A mini-review. Frontiers in Psychology.

[B71-behavsci-15-00593] Webster G. D., DeWall C. N., Pond R. S., Deckman T., Jonason P. K., Le B. M., Nichols A. L., Schember T. O., Crysel L. C., Crosier B. S., Smith C. V., Paddock E. L. (2015). The brief aggression questionnaire: Structure, validity, reliability, and generalizability. Journal of Personality Assessment.

[B72-behavsci-15-00593] Webster G. D., DeWall C. N., Pond R. S., Deckman T., Jonason P. K., Le B. M., Nichols A. L., Schember T. O., Crysel L. C., Crosier B. S., Smith C. V., Paddock E. L., Nezlek J. B., Kirkpatrick L. A., Bryan A. D., Bator R. J. (2014). The brief aggression questionnaire: Psychometric and behavioral evidence for an efficient measure of trait aggression. Aggressive Behavior.

[B73-behavsci-15-00593] Williams J. M., Straub W. F., Williams J. M. (2006). Sport psychology: Past, present, future. Applied sport psychology: Personal growth to peak performance.

[B74-behavsci-15-00593] Winsler A., Feder A., Way E. L., Manfra L. (2006). Maternal beliefs concerning young children’s private speech. Infant and Child Development.

[B75-behavsci-15-00593] Young A. D., Hollander D. B., Baiamonte B. A., Bowers A., Hebert E. P., Kraemer R. R. (2023). How much does self-talk influence fatigue? A comparison of performance, perceived exertion, and neuromuscular patterns during high-intensity power cleans: Self-talk and fatigue. International Journal of Strength and Conditioning.

